# Efficacy of Combination Therapy With Sitagliptin and Low-Dose Glimepiride in Japanese Patients With Type 2 Diabetes

**DOI:** 10.14740/jocmr1701w

**Published:** 2014-02-06

**Authors:** Hiroaki Ishii, Yosuke Ohkubo, Masahiro Takei, Shinichi Nishio, Masanori Yamazaki, Mieko Kumagai, Yoshihiko Sato, Satoru Suzuki, Yuji Aoki, Takahide Miyamoto, Tomoko Kakizawa, Takahiro Sakuma, Mitsuhisa Komatsu

**Affiliations:** a Department of Internal Medicine, Division of Diabetes, Endocrinology and Metabolism, Shinshu University School of Medicine, Japan; b National Hospital Organization Matsumoto Medical Center, Japan; c Miyamoto Medical Clinic, Japan; d Kakizawa Medical Clinic, Japan; e Ina Central Hospital, Japan

**Keywords:** Sitagliptin, Glimepiride, Combination therapy, Sulfonylurea, Dipeptidyl peptidase (DPP)-4 inhibitors, Type 2 diabetes mellitus, Antihyperglycemicagents, Hypoglycemia

## Abstract

**Background:**

We examined the effects of combination therapy with 50 mg/day of sitagliptin and low-dose glimepiride (1 mg/day) in patients with type 2 diabetes.

**Methods:**

Twenty-six patients with poorly controlled type 2 diabetes currently taking high-dose glimepiride (≥ 2 mg/day) were enrolled in the study. The dose of glimepiride was reduced to 1 mg/day and 50 mg/day of sitagliptin was added without changing the doses of any other antihyperglycemic agents. The patients were divided into two groups: the low-dose group (2 or 3 mg glimepiride decreased to 1 mg: n = 15) and the high-dose group (4 or 6 mg glimepiride decreased to 1 mg: n = 11).

**Results:**

Combination therapy significantly lowered HbA1c after 24 weeks of treatment in both groups. In the low-dose group, 8.1 ± 0.2% decreased to 7.0 ± 0.1%; in the high-dose group, 8.4 ± 0.1% decreased to 7.3 ± 0.2%. The time course of the degree of HbA1c reduction in the high-dose group was almost superimposable on that in the low-dose group. There were no changes in body weight and no hypoglycemia and in either group during the study period. In conclusion, our results suggested that the combination therapy used in the study is both well tolerated and effective.

**Conclusion:**

This study indicated the usefulness of dipeptidyl peptidase (DPP)-4 inhibitors in Japanese patients with type 2 diabetes, and also reinforces the importance of low doses of sulfonylurea for effective glycemic management.

## Introduction

Dipeptidyl peptidase (DPP)-4 inhibitors have emerged as a new category of oral hypoglycemic agents for type 2 diabetes, which are widely used worldwide [1]. In the position statement of the American Diabetes Association and the European Association for the Study of Diabetes, metformin is the first drug of choice for type 2 diabetes, and DPP-4 inhibitors are cited as one of second-line drugs [2]. In Japan, all classes of oral hypoglycemic agents may be included as first-line drugs depending on individual pathophysiological features [3]. In particular, sulfonylurea (SU), an insulinotropic agent, is often chosen based on the relative importance of insulin deficiency in Japanese type 2 diabetes patients. DPP-4 inhibitors increase plasma GLP-1 concentration and elevate cellular cAMP levels in pancreatic beta-cells leading to potentiate insulin secretion, whereas SU stimulates insulin secretion as a result of elevation of cytosolic Ca^2+^ concentration in the beta-cells [4]. Therefore, it is anticipated that a combination of DPP-4 inhibitor and SU may synergistically stimulate insulin secretion and effectively ameliorate hyperglycemia in type 2 diabetes mellitus.

However, cases of serious hypoglycemia due to the combination of SU and sitagliptin, a DPP-4 inhibitor, accumulated over the first several months after launching sitagliptin in Japan [5]. In response to this situation, the Japan Association for Diabetes Education and Care recommended that the dose of glimepiride should be decreased to at 2 mg/day to avoid hypoglycemia when DPP-4 is added to the regimen in patients treated with higher doses of glimepiride [6]. Our preliminary clinical experience suggested that the dose of glimepiride could be decreased to 1 mg/day without reducing its efficacy given 50 mg/day sitagliptin is added. To test this hypothesis, we performed a prospective observational study to examine the effects of combination therapy of sitagliptin and low-dose glimepiride for poorly controlled type 2 diabetes, despite treatment with various doses of glimepiride.

## Materials and Methods

The study population consisted of 26 patients with poorly controlled type 2 diabetes who were taking a high dose of glimepiride (≥ 2 mg/day) at several hospitals or clinics in Nagano Prefecture, Japan. Exclusion criteria were patients with type 1 diabetes or type 2 diabetes taking insulin therapy, significant renal impairment (serum creatinine > 1.5 mg/dL), proliferative diabetic retinopathy, apparent cardiac disease (New York Heart Association grade > II), or anemia (hemoglobin ≤ 11.0 g/dL). Type 1 diabetes was excluded by examination of autoantibodies to glutamic acid decarboxylase. We decreased the dose of glimepiride to 1 mg/day and added 50 mg/day of sitagliptin without changing the doses of any other antihyperglycemic agents. The patients were divided into two groups: the low-dose group (glimepiride dose was decreased from 2 or 3 mg to 1 mg, n = 15) and the high-dose group (glimepiride dose was decreased from 4 or 6 mg to 1 mg, n = 11). HbA1c, casual plasma glucose, body weight, and subjective symptoms were checked monthly in each group for 6 months. HbA1c values were converted from JDS to NGSP values by the conversion formulas [7, 8].

All patients provided informed consent to participation in the study. The study protocol was approved by the ethics committee of Shinshu University School of Medicine. The study was performed in accordance with the ethical principles of the Declaration of Helsinki amended in Edinburgh in 2000.

All data are presented as means ± SEM. For statistical analysis, paired t-test and Wilcoxon’s rank sum test were performed. In all analyses, P < 0.05 was taken to indicate statistical significance.

## Results

The basal characteristics of the patients are shown in Table 1. The combination of 1 mg of glimepiride and 50 mg of sitagliptin significantly lowered HbA1c after 24 weeks of treatment in both low- and high-dose glimepiride groups.

**Table 1:** Patient Background

	All	High dose group	Low dose group
Number (male:female)	26 (17:9)	11 (6:5)	15 (11:4)
Dose of glimepiride		4 mg or 5 mg or 6 mg	2 mg or 3 mg
Age (year)	67.1 ± 1.7	70.3 ± 2.2*	62.8 ± 3.1
Body weight (kg)	66.6 ± 2.2	62.1 ± 2.6*	69.6 ± 3.0
HbAlc (%)	8.2 ± 0.1	8.4 ± 0.1*	8.1 ± 0.2
Casual plasma glucose (mg/dL)	194 ± 10	211 ± 21	182 ± 8

Mean ± SEM, *P < 0.05 vs. Low dose group.

**Figure 1:**
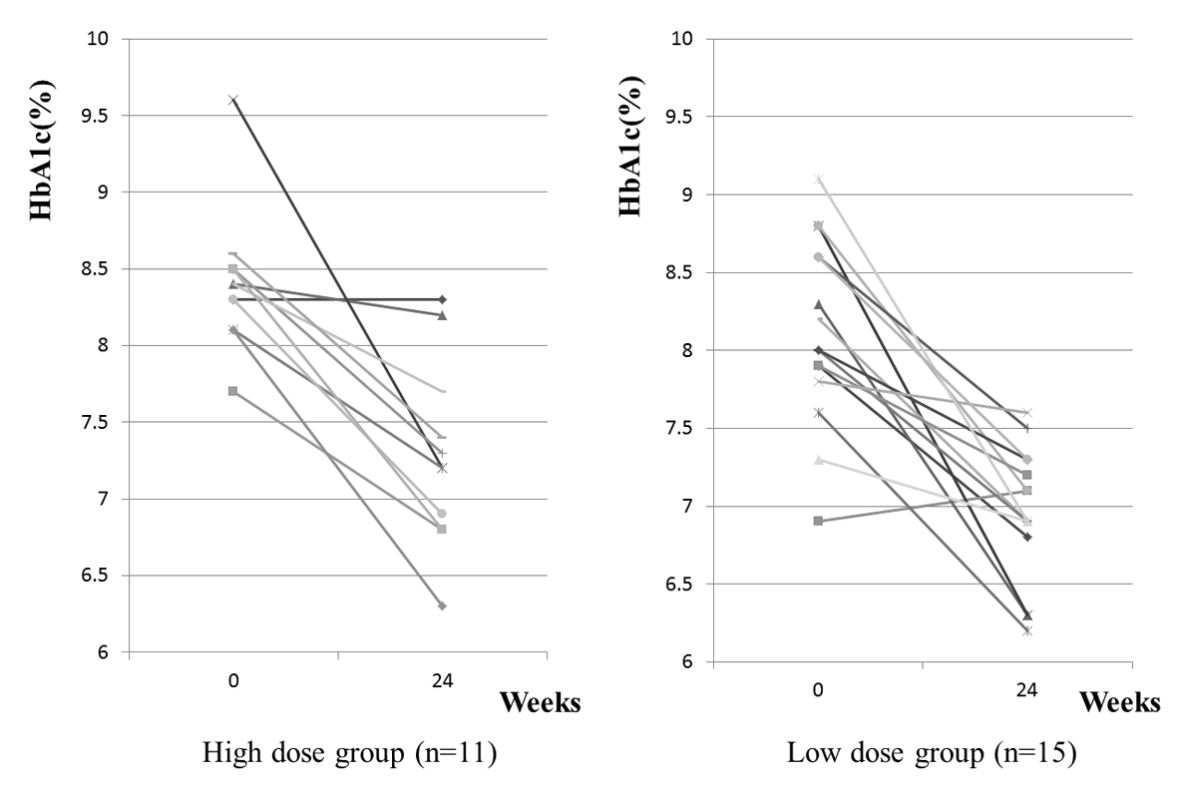
HbA1c of each patientbefore and after 24 weeks. Left and right panel shows results of high dose and low dose groups, respectively. After 24 weeks of the combination of 1 mg/day glimepiride and 50 mg/day sitagliptin, all subjects except one case in the low dose group exhibit improvement of HbA1c.

As shown in Figure 1, HbA1c decreased from 8.1 ± 0.2% to 7.0 ± 0.1% in the low-dose group and from 8.4 ± 0.1% to 7.3 ± 0.2% in the high-dose group (reductions of 1.1% and 1.1%, respectively). Although HbA1c at registration was slightly higher in the high-dose group (Table 1), combination therapy was similarly effective in both groups. HbA1c worsened after 24 weeks of treatment in only 1 of the 26 patients.

**Figure 2:**
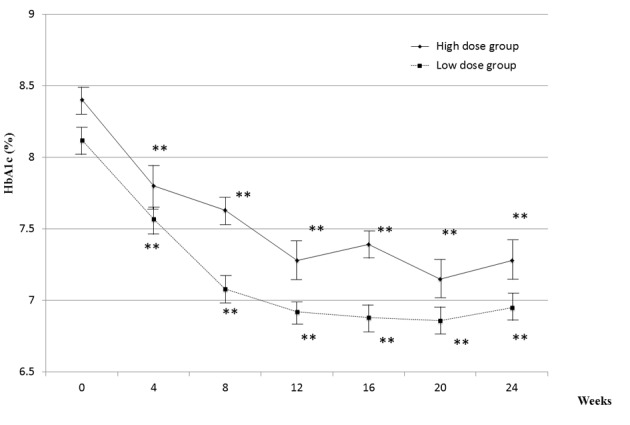
Changes in HbA1c for 24 weeks. Actual mean values of HbA1c in two groups are shown. The values are mean ± SEM. A statistical analysis was performed by Paired t-test.* *P < 0.01 vs. 0 week.

**Figure 3:**
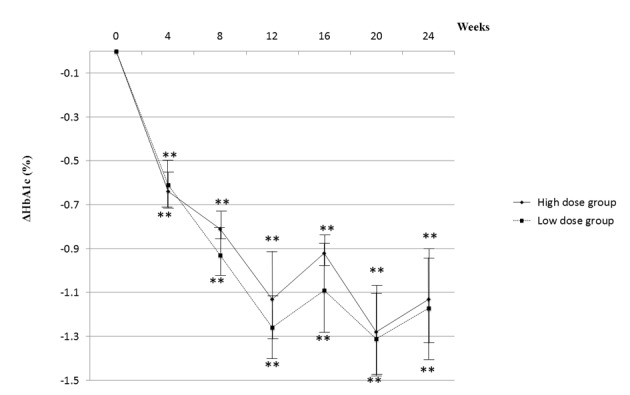
Degrees of HbA1c reduction are shown. A statistical analysis was performed by the Wilcoxson rank sum test, paired t-test.* *P < 0.01 vs. 0 week.

Figure 2 showed changes in HbA1c over the 24-week treatment period. All patients received 1 mg of glimepiride and 50 mg of sitagliptin with fixed doses of other hypoglycemic agents. With the combination therapy, HbA1c decreased sharply within 12 weeks in both low- and high-dose groups. The lowering effect of the treatment lasted for 24 weeks. As shown in Figure 3, the time course of the change in degree of HbA1c reduction in the high-dose group was almost superimposable on that in the low-dose group.

Throughout the study, there was no severe hypoglycemia in either group. None of the patients even showed mild hypoglycemic symptoms, although they were asked to report symptoms of hypoglycemia, such as palpitation, sweating, or unusual feelings of hunger, at each visit. Two patients complained of temporary abdominal fullness, but treatment was continued in both cases. Body weight remained unchanged in both groups during the study period (69.6 ± 3.0 kg to 69.1 ± 2.9 kg in the low-dose group, 62.1 ± 2.6 kg to 61.9 ± 3.0 kg in the high-dose group, both P > 0.05).

**Figure 4:**
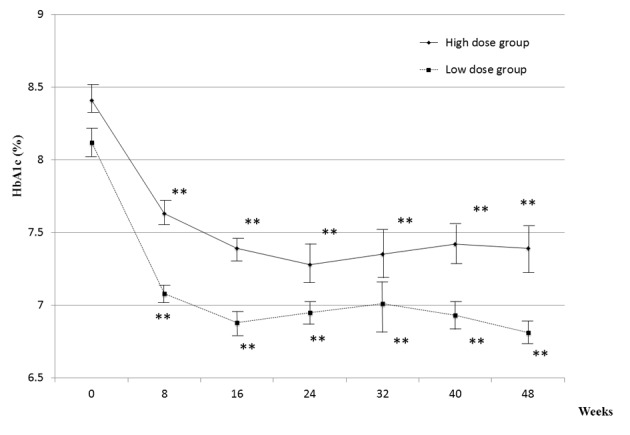
Changes in HbA1c for 48 weeks. Actual mean values of HbA1c in two groups are shown. The values are mean ± SEM. A statistical analysis was performed by Paired t-test.* *P < 0.01 vs. 0 week.

All patients continued the treatment for 48 weeks with the same medication except in 4 cases; 1 patient required an increase in glimepiride dose to 2 mg/day and 3 patients required an increase in sitagliptin dose to 100 mg/day. The time courses of changes in HbA1c for 48 weeks are shown in Figure 4. These observations indicated that the efficacy of combination therapy lasted for 48 weeks.

## Discussion

In this study, we demonstrated that the combination of 1 mg of glimepiride and 50 mg of sitagliptin ameliorated glycemic control in Japanese type 2 diabetes patients irrespective of prior dose of glimepiride. As no hypoglycemia was reported during the study, this combination is both safe and effective for the treatment of type 2 diabetes.

Despite a decrease in the dose of glimepiride, the mean HbA1c was reduced from 8.2% to 7.1% after 24 weeks of treatment with addition of sitagliptin. A decline of 1.05% was reported in a phase III trial of sitagliptin monotherapy in Japanese type 2 diabetes patients [9]. These observations indicate that combination therapy with SU and DPP4 inhibitor, such as sitagliptin, is useful even when SU monotherapy failed to ameliorate hyperglycemia. The efficacy of the combination therapy may have been because most of our patients (18 of 26 patients) were on biguanide, which was continued during the study period. There are several potential mechanisms for the synergistic effect of biguanide with DPP4 inhibitor. Biguanide promotes GLP-1 secretion from intestinal L cells [10, 11], inhibits DPP4 activity [12], and reduces the effect of glucagon, which is also suppressed by GLP-1, in the liver [13].

It is intriguing that we obtained similar therapeutic effects with 1 mg of glimepiride irrespective of the previous dose of glimepiride. Administration of 1 mg of glimepiride may be sufficient to increase intracellular calcium concentration in the beta-cells to the level required to induce insulin secretion. This is compatible with the notion that SU should be used at low doses to prevent hypoglycemia due to inappropriate insulin secretion. As SU is known to stimulate glucagon secretion [14], the reduced SU dose may work in favor of the robust hypoglycemic effect.

Glucose stimulates insulin secretion from pancreatic beta-cells via KATP channel-dependent and -independent pathways [4]. SU mimics the glucose-induced KATP channel-dependent pathway. Although the molecular mechanisms underlying the KATP channel-independent pathway remain unknown [15], an increase in cAMP induced by incretin therapy potentiates KATP channel-independent insulinotropic action by glucose [16, 17]. Therefore, SU and DPP4 inhibitor may be an effective combination for supporting inappropriate insulin secretion in type 2 diabetes.

There were several limitations of our study. First, the number of cases was small. Although the purpose of this study was to explore the potential efficacy and safety of the combination therapy, further studies in larger populations are required to confirm our conclusions. The patients were divided into two groups (high-dose and low-dose) depending on the amount of glimepiride administered before the combination therapy, and a comparison was performed between the groups. As this was a prospective observational study, it will be necessary to include control group in which sitagliptin is added without changing the amount of glimepiride. This will allow a discussion of the safety and utility of this combination therapy. However, from an ethical standpoint this would be difficult to do [6]. With regard to the expression of hypoglycemia, at each visit we carefully interviewed to the patients regarding whether they experienced hypoglycemic symptoms; however, some patients may have been unaware of hypoglycemic symptoms.

In conclusion, our results suggest that the combination therapy used in this study is well tolerated and effective. The favorable results of the combination therapy with DPP4 inhibitor and low-dose SU may be because most patients were treated with biguanide before and after the study. However, it is also possible that the combination therapy effectively or synergistically stimulated insulin secretion in our patients. This study suggested the usefulness of DPP4 inhibitors in Japanese patients with type 2 diabetes, and also reinforces the importance of low-dose SU for effective glycemic management.
